# Supplementary material for: Biosynthesis of the antibiotic nonribosomal peptide penicillin in baker's yeast
Source: Nat Commun. 2017 May 4;8:15202. doi: 10.1038/ncomms15202 (PMC5418595; doi:10.1038/ncomms15202)
Supplement: Supplementary Information — Supplementary figures and supplementary tables. [file ncomms15202-s1.pdf]

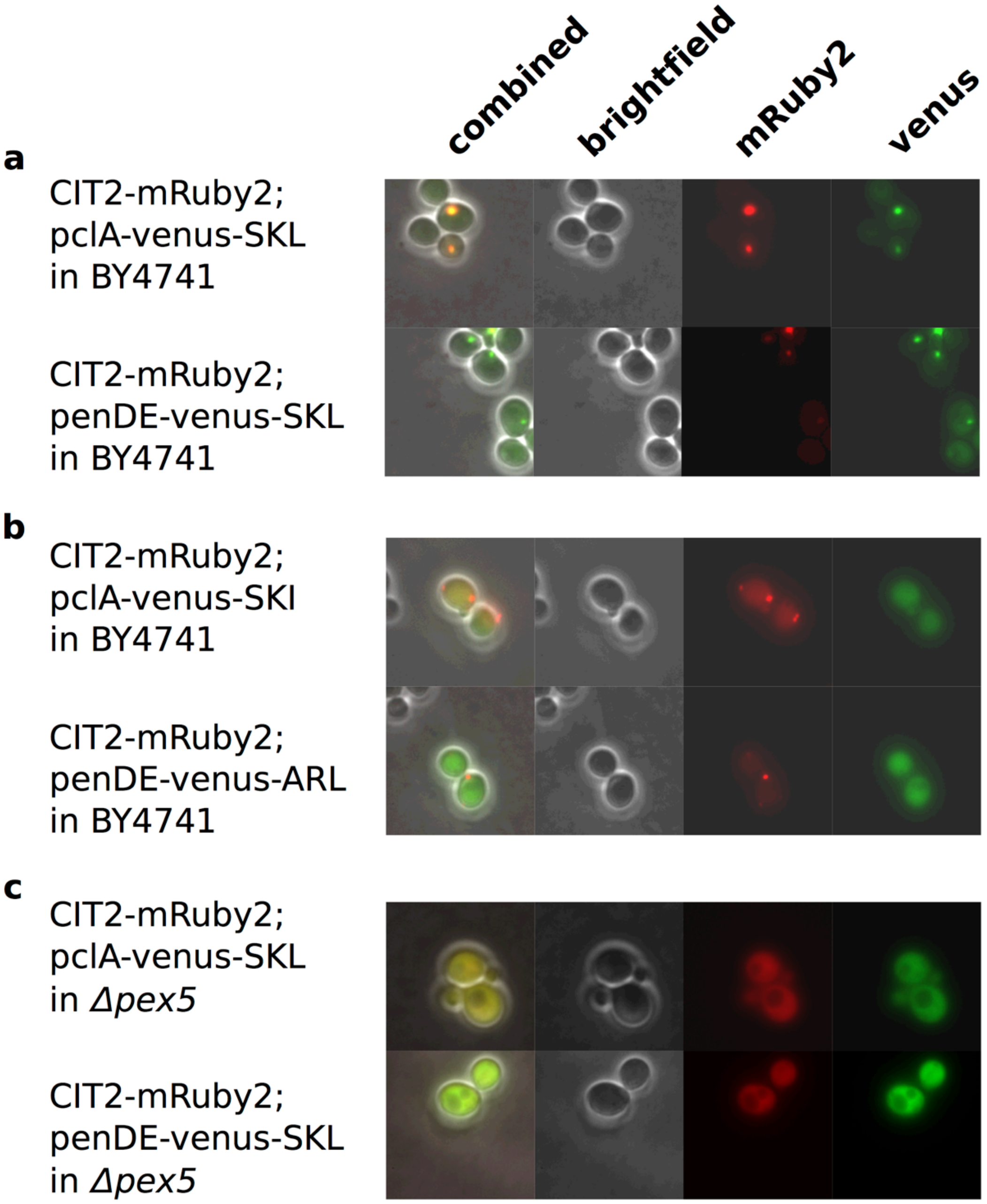


**Supplementary Figure 1. *S. cerevisiae* PTS1 tags are necessary to localise pclA and penDE to peroxisomes. (a)** *S. cerevisiae* peroxisomal protein CIT2 with an mRuby2 fluorescence tag co-localises with venus-fluorescence-tagged *pclA* and *penDE* proteins additionally tagged with the *S. cerevisiae* Peroxisome Targeting Sequence 1 (PTS1) tripeptide SKL at the C-terminus. For this and subsequent parts, the promoter driving expression of *pclA* and *penDE* with the venus fluorescence tag is the strong constitutive promoter pTDH3. **(b)** Venus-fluorescence-tagged *pclA* and *penDE* proteins without the *S. cerevisiae* PTS1 but tagged with the native *P. chrysogenum* PTS1 tripeptides SKI and ARL do not co-localise with mRuby2-tagged CIT2. **(c)** Plasmids from part (a) transformed into an *S. cerevisiae* strain harbouring a delete of the peroxisomal importer *pex5* prevent peroxisomal localisation of CIT2 and the *S. cerevisiae* PTS1-tagged *pclA* and *penDE* proteins.

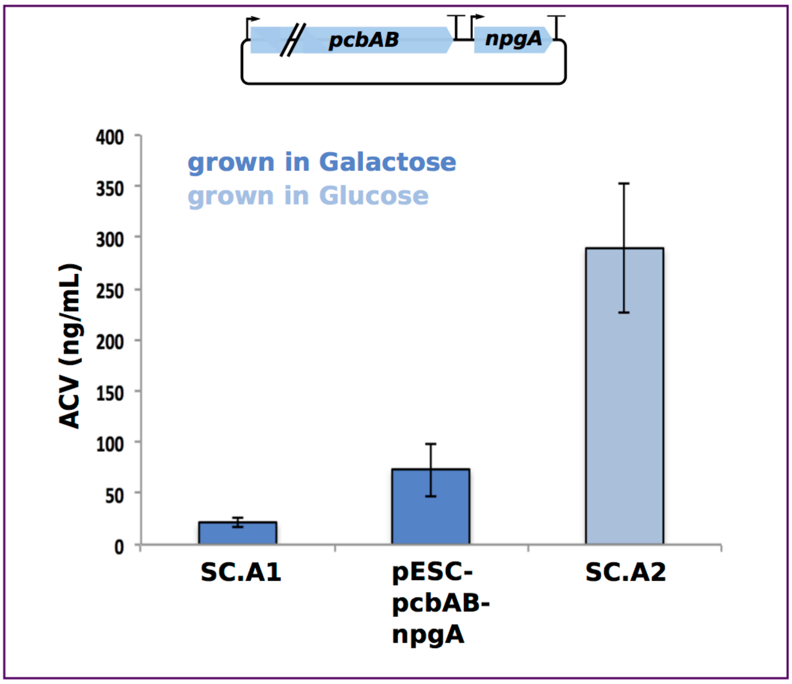


**Supplementary Figure 2. Optimising ACV yields.** Strong, constitutive promoters driving the ACV-producing part of the benzylpenicillin pathway on a low-copy plasmid (Sc.A2) gives higher maximal ACV yields than the same genes under galactose inducible promoters either integrated into a chromosome (Sc.A1) or on a high-copy plasmid (pESC-pcbAB-npgA). Sc.A2 was grown using glucose as the carbon source, while Sc.A1 and pESC-pcbAB-npgA were grown using galactose as the carbon source. Error bars represent standard deviation from three biological replicates.

| pcbAB | npgA | GAL 1 | GAL 2 | GAL 3 | mean GAL | GAL s.d. | Glu 1 | Glu 2 | Glu 3 | mean GLU | Glu s.d. |
|---|---|---|---|---|---|---|---|---|---|---|---|
| GAL1 | GAL7 | 502212.0 | 633534.0 | 217523.0 | 451089.7 | 212665.0 | Not detected | Not detected | Not detected | 0.0 | 0.0 |
| GAL1 | TDH3 | 786826.0 | 943928.0 | 391730.0 | 707494.7 | 284518.5 | Not detected | Not detected | Not detected | 0.0 | 0.0 |
| GAL1 | HHF2 | 561543.0 | 649935.0 | 279805.0 | 497094.3 | 193298.4 | Not detected | Not detected | Not detected | 0.0 | 0.0 |
| GAL1 | RPL18B | 545298.0 | 565738.0 | 349162.0 | 486732.7 | 119577.2 | Not detected | Not detected | Not detected | 0.0 | 0.0 |
| RPL18B | GAL1 | 100022.0 | 116057.0 | 51593.0 | 89224.0 | 33561.1 | 56568.0 | 53651.0 | 43446.0 | 51221.7 | 6890.1 |
| RPL18B | PGK1 | 138596.0 | 110451.0 | 58385.0 | 102477.3 | 40695.6 | 197598.0 | 109863.0 | 157372.0 | 154944.3 | 43917.9 |
| RPL18B | CCW12 | 144395.0 | 116449.0 | 59786.0 | 106876.7 | 43109.1 | 211374.0 | 150211.0 | 87174.0 | 149586.3 | 62102.4 |
| RPL18B | ALD6 | 146092.0 | 68694.0 | 56735.0 | 90507.0 | 48508.0 | 131432.0 | 100424.0 | 107881.0 | 113245.7 | 16185.1 |
| TDH3 | PGK1 | 193113.0 | 86218.0 | 103581.0 | 127637.3 | 57364.3 | 404773.0 | 277704.0 | 284469.0 | 322315.3 | 71490.5 |
| TDH3 | CCW12 | 178619.0 | 138064.0 | 73651.0 | 130111.3 | 52934.0 | 361186.0 | 331388.0 | 218401.0 | 303658.3 | 75323.2 |
| TDH3 | ALD6 | 168824.0 | 74255.0 | 60426.0 | 101168.3 | 58998.1 | 369769.0 | 201340.0 | 218023.0 | 263044.0 | 92802.2 |
| HHF2 | PGK1 | 87157.0 | 40076.0 | 36120.0 | 54451.0 | 28393.2 | 56687.0 | 40722.0 | 28941.0 | 42116.7 | 13925.5 |
| HHF2 | CCW12 | 76374.0 | 72364.0 | 46495.0 | 65077.7 | 16217.5 | 34133.0 | 42551.0 | 21065.0 | 32583.0 | 10826.5 |
| HHF2 | ALD6 | 84088.0 | 32408.0 | 24310.0 | 46935.3 | 32428.9 | 65046.0 | 31137.0 | 45320.0 | 47167.7 | 17029.8 |

**ACV Yields**

| Strain | pcbAB | npgA | sugar | reading 1 | reading 2 | reading 3 | mean conc. (ng/uL) | conc. s.d. |
|---|---|---|---|---|---|---|---|---|
| Sc.A1 | GAL10 | GAL1 | gal | 26.868 | 20.011 | 17.482 | 21.454 | 4.857 |
| pESC-npgA-pcbAB | GAL10 | GAL1 | gal | 102.938 | 53.643 | 63.204 | 73.262 | 26.142 |
| Sc.A2 | TDH3 | PGK1 | glu | 362.586 | 249.952 | 255.949 | 289.495 | 63.369 |

**Supplementary Table 1. LCMS peak areas and yields for ACV for the pcbAB, npgA promoter screen**

| Name (X = sample from screened strain) | Area |
|---|---|
| Water | |
| Water | |
| benzylpenicillin; 10 pg/uL | |
| benzylpenicillin; 100 pg/uL | 2233 |
| benzylpenicillin; 1 ng/uL | 18103 |
| benzylpenicillin; 10 ng/uL | 165306 |
| Water | 981629 |
| Water | |
| X1 | |
| X2 | |
| X3 | |
| X4 | |
| X5 | |
| X6 | |
| X7 | |
| X8 | |
| X9 | |
| X10 | |
| X11 | |
| X12 | 318 |
| X13 | |
| X14 | |
| X15 | |
| X16 | |
| X17 | |
| X18 | |
| X19 | 2761 |
| X20 | |
| X21 | 2709 |
| X22 | 1062 |
| X23 | |
| X24 | |
| X25 | |
| X26 | |
| X27 | 1292 |
| X28 | |
| X29 | |
| X30 | |
| X31 | |
| X32 | |
| X33 | |
| X34 | |
| X35 | |
| X36 | |
| X37 | 527 |
| X38 | |
| X39 | |
| X40 | |
| X41 | |
| X42 | |
| X43 | 359 |
| X44 | |
| X45 | |
| X46 | |
| X47 | |
| X48 | |

| Name (X = sample from screened strain) | Area |
|---|---|
| X49 | |
| X50 | |
| X51 | |
| X52 | |
| X53 | |
| X54 | |
| X55 | |
| X56 | |
| X57 | 1506 |
| X58 | |
| X59 | |
| X60 | |
| X61 | |
| X62 | |
| X63 | |
| X64 | |
| X65 | |
| X66 | |
| X67 | |
| X68 | |
| X69 | |
| X70 | |
| X71 | 385 |
| X72 | |
| X73 | 1285 |
| X74 | |
| X75 | |
| X76 | 295 |
| X77 | |
| X78 | |
| X79 | |
| X80 | 411 |
| X81 | |
| X82 | |
| X83 | |
| X84 | 665 |
| X85 | |
| X86 | |
| X87 | |
| X88 | |
| X89 | |
| X90 | 467 |
| X91 | |
| X92 | |
| X93 | |
| X94 | |
| X95 | |
| X96 | |
| X97 | |
| X98 | 2193 |
| X99 | |
| X100 | |
| X101 | |
| X102 | |
| X103 | |
| X104 | |

| Name (X = sample from screened strain) | Area |
|---|---|
| X105 | |
| X106 | |
| X107 | |
| X108 | |
| X109 | |
| X110 | |
| X111 | |
| X112 | |
| X113 | |
| X114 | |
| X115 | |
| X116 | |
| X117 | |
| X118 | 586 |
| X119 | |
| X120 | |
| X121 | |
| X122 | |
| X123 | |
| X124 | |
| X125 | |
| X126 | |
| X127 | |
| X128 | |
| X129 | |
| X130 | |
| X131 | |
| X132 | |
| X133 | |
| X134 | |
| X135 | |
| X136 | |
| X137 | |
| X138 | 171 |
| X139 | |
| X140 | |
| X141 | |
| X142 | |
| X143 | |
| X144 | |
| X145 | |
| X146 | 692 |
| X147 | |
| X148 | 1648 |
| X149 | |
| X150 | 1060 |
| X151 | |
| X152 | |
| X153 | |
| X154 | |
| X155 | |
| X156 | |
| X157 | |
| X158 | |
| X159 | |
| X160 | |

**Supplementary Table 2. LCMS peak areas and yields for ACV for the pcbAB, npgA promoter screen**

**Summed nanopore read count data for single assemblies**

| | pcbC | pclA | penDE |
|---|---|---|---|
| pGAL1 | 58 | 79 | 25 |
| pGAL7 | 216 | 139 | 33 |
| pTDH3 | 40 | 107 | 33 |
| pCCW12 | 39 | 59 | 42 |
| pPGK1 | 209 | 89 | 32 |
| pHHF2 | 317 | 91 | 52 |
| pRPL18B | 105 | 71 | 39 |
| pALD6 | 190 | 125 | 42 |
| pPSP2 | 103 | 44 | 73 |
| pREV1 | 78 | 67 | 26 |
| *TOTAL* | *1355* | *871* | *397* |

**Summed nanopore read count data for multiegene level assembly**

| | pcbC | pclA | penDE |
|---|---|---|---|
| pGAL1 | 16 | 15 | 12 |
| pGAL7 | 19 | 24 | 7 |
| pTDH3 | 12 | 10 | 10 |
| pCCW12 | 17 | 11 | 13 |
| pPGK1 | 7 | 28 | 13 |
| pHHF2 | 17 | 27 | 25 |
| pRPL18B | 21 | 4 | 12 |
| pALD6 | 3 | 3 | 14 |
| pPSP2 | 13 | 5 | 26 |
| pREV1 | 10 | 8 | 3 |
| *TOTAL* | *135* | *135* | *135* |

**Summed Sanger read count data from all 10 benzylpenicillin producers**

| | pcbC | pclA | penDE |
|---|---|---|---|
| pGAL1 | 0 | 0 | 0 |
| pGAL7 | 1 | 0 | 0 |
| pTDH3 | 0 | 2 | 2 |
| pCCW12 | 1 | 5 | 0 |
| pPGK1 | 0 | 3 | 2 |
| pHHF2 | 2 | 0 | 2 |
| pRPL18B | 5 | 0 | 1 |
| pALD6 | 0 | 0 | 0 |
| pPSP2 | 1 | 0 | 3 |
| pREV1 | 0 | 0 | 0 |
| *TOTAL* | *10* | *10* | *10* |

**one-sided Fisher's exact test for enrichment of pclA strong promoters in multigene assemblies vs. benzylpenicillin producers**

| | strong | non-strong |
|---|---|---|
| **multigene** | 44 | 938 |
| **producers** | 10 | 0 |

**p < 0.005** (Fisher's exact test)

**Observed nanopore read count data by promoter category for single assemblies**

| | pcbC | pclA | penDE |
|---|---|---|---|
| **Strong** | 605 | 346 | 159 |
| **Medium** | 295 | 196 | 81 |
| **Weak** | 181 | 111 | 99 |
| **Inducible** | 274 | 218 | 58 |

**Expected nanopore read counts for single assemblies for Chi square test based on promoter proportions used in library construction**

| | pcbC | pclA | penDE |
|---|---|---|---|
| **Strong** | 542 | 348 | 159 |
| **Medium** | 271 | 174 | 79 |
| **Weak** | 271 | 174 | 79 |
| **Inducible** | 271 | 174 | 79 |

**Observed nanopore read count data by promoter category for multigene assemblies**

| | pcbC | pclA | penDE |
|---|---|---|---|
| **Strong** | 53 | 76 | 61 |
| **Medium** | 24 | 7 | 26 |
| **Weak** | 23 | 13 | 29 |
| **Inducible** | 35 | 39 | 19 |

**Expected nanopore read count data by promoter category for multigene assemblies based on single assembly observed distributions**

| | pcbC | pclA | penDE |
|---|---|---|---|
| **Strong** | 60 | 54 | 54 |
| **Medium** | 29 | 30 | 28 |
| **Weak** | 18 | 17 | 34 |
| **Inducible** | 27 | 34 | 20 |

**Observed nanopore read count data by promoter category for producers**

| | pcbC | pclA | penDE |
|---|---|---|---|
| **Strong** | 3 | 10 | 6 |
| **Medium** | 5 | 0 | 1 |
| **Weak** | 1 | 0 | 3 |
| **Inducible** | 1 | 0 | 0 |

**p-values for Pearson's Chi-squared tests comparing single to multigene level assembly, and Fisher's exact test comparing multigene level assembly to producer for promoter category distributions**

| | pcbC | pclA | penDE |
|---|---|---|---|
| **uniform dist vs. single** | p < 0.27 | p < 0.27 | p < 0.27 |
| **single vs. multigene** | p < 0.22 | p < 0.22 | p < 0.22 |
| **multigene vs. producers** | p < 0.15 | p < 0.005 | p < 0.52 |

## Supplementary Table 3. Read counts and statistics for nanopore and Sanger sequencing

| Experiment | Strain name(s) | Background Strain | plasmid(s) |
|---|---|---|---|
| Fig 1c, 2a | Sc.A1 | BY4741 | - |
| Fig 1c, d | Sc.P1 | Sc.A1 | pAA056 |
| Fig 1c | Sc.P1x | | |
| Fig 2b, 3 | Sc.P2 | Sc.A2 | pAA179 |
| Fig 3 | Sc.P2x | BY4741 | pAA180, pAA171 |
| Fig 3 | Sc.S1 – Sc.S12 | BY4741 | variants of pAA179* |
| Fig S1 | - | BY4741 | pAA210 |
| Fig S1 | - | BY4741 | pAA211 |
| Fig S1 | - | BY4741 | pAA212 |
| Fig S1 | - | BY4741 | pAA213 |
| Fig S1 | - | BY4741*Δpex5* | pAA210 |
| Fig S1 | - | BY4741*Δpex5* | pAA211 |
| Fig S2 | pESC-npgA-pcbAB | BY4741 | pESC-npgA-pcbAB |
| Fig S2 | Sc.A2 | BY4741 | pAA145 |

**Primers used to create the *Δpex5* strain**

| Purpose | Orientation | Sequence |
|---|---|---|
| to amplify and retarget gRNA vector | F | CGTTTCTATTTTGAATACTAaaagtcccattcgccacccg |
| to amplify and retarget gRNA vector | R | /5Phos/gttttagagctagaaatagcaagttaaaataaggctag |
| Overlap extension PCR 1 | F | GTGAAGGAAATCGCAGCTCC |
| Overlap extension PCR 1 | R | AATGCTAATGAATTTGGGCAGTGATGCGAGCTCCGCCGGAAGAGGGTACCGACTTGTCGG |
| Overlap extension PCR 2 | F | CCGACAAGTCGGTACCCTCTTCCGGCGGAGCTCGCATCACTGCCCAAATTCATTAGCATT |
| Overlap extension PCR 2 | R | CCTCTCTGTATTCGTGAGAGTATC |

**Supplementary Table 4. Strains and plasmids used in this paper**
* same as pAA179, but with promoters driving pcbC, pclA and penDE as specified by Table S12

| Experiment | Molecule of interest | Sugar | Dropouts | Supplements | Volume |
|---|---|---|---|---|---|
| Fig 1c, d | ACV | galactose | uracil | 5 mM AAA | 30 mL |
| Fig 1c, d | benzylpenicillin | glucose | uracil, histidine | 5 mM AAA, 0.25 mM PAA | 30 mL |
| Fig S2 | ACV | galactose/ glucose | uracil | 5 mM AAA | 600 μL |
| Fig 2b, 3, S3, S4 | benzylpenicillin | glucose | uracil, histidine | 5 mM AAA, 0.25 mM PAA | 600 μL |

AAA = alpha-amino adipic acid (VWR L13924.0
PAA = phenylacetic acid (Sigma P16621)

**Supplementary Table 5. ACV and benzylpenicillin production media composition**

**The LC gradient elution method for the separation of compounds in the penicillin-G pathway**

| Time (minutes) | % Solvent A | % Solvent B | Flow rate (mL/min) |
|---|---|---|---|
| 0 | 98 | 2 | 0.2 |
| 2 | 98 | 2 | 0.2 |
| 8 | 35 | 65 | 0.2 |
| 8.01 | 5 | 95 | 0.2 |
| 9 | 5 | 95 | 0.2 |

**The MS/MS fragment ions used for the measurement of benzylpenicillin and other materials in the pathway**

| Analyte | Formula | Precursor ion [M+H]+ | Product ion | Collision energy (eV) |
|---|---|---|---|---|
| benzylpenicillin | C16H18N2O4S | 335.106 | 176.0707 | 16 |
| LLD-ACV | C14H25N3O6S | 364.1537 | 144.0655 | 16 |

**Supplementary Table 6. LCMS specifications**

| Promoter | Category | Relative Expression* |
|---|---|---|
| pTDH3 | Strong | 820 |
| pCCW12 | Strong | 575 |
| pPGK1 | Strong | 291 |
| pHHF2 | Strong | 230 |
| pRPL18B | Medium | 60 |
| pALD6 | Medium | 39 |
| pPSP2 | Weak | 2.2 |
| pREV1 | Weak | 1.7 |
| pGAL1 | Inducible | 1.3 |
| pGAL7 | Inducible | ** |

**Supplementary Table 7. Promoters used in first promoter screen to optimise conversion of ACV to benzylpenicillin.**

* Based on fold-change expression over background of fluorescent reporter Venus protein under each promoter, cells grown in glucose.
Taken from Lee, M.E., DeLoache, W.C., Cervantes, B. & Dueber, J.E.
ACS Synth Biol 4, 975-986 (2015).

** Uncharacterised in the above study, but should be similar to value from pGAL1, as these promoters naturally drive similar levels of expression when uninduced.

| Sample | pcbC | pclA | penDE | LCMS | pclA promoter Strength | penDE promoter Strength | Geometric mean of promoter strength pclA + penDE | [benzylpenicillin] ng/mL |
|---|---|---|---|---|---|---|---|---|
| X19 | pRPL18B | pTDH3 | pCCW12 | 2761 | 820 | 575 | 686.659 | 3.204 |
| X148 | pRPL18B | pTDH3 | pPGK1 | 1648 | 820 | 291 | 488.487 | 1.845 |
| X98 | pRPL18B | pCCW12 | pPGK1 | 2193 | 575 | 291 | 409.054 | 2.505 |
| X80 | pRPL18B | pTDH3 | pGAL1 | 411 | 820 | 1.3 | 32.650 | 0.418 |
| X84 | pRPL18B | pPGK1 | pPGK1 | 665 | 291 | 291 | 291.000 | 0.699 |
| X37 | pRPL18B | pPGK1 | pHHF2 | 527 | 291 | 230 | 258.708 | 0.545 |
| X90 | pRPL18B | pALD6 | pHHF2 | 467 | 39 | 230 | 94.710 | 0.479 |

**Supplementary Table 8. Relationship between promoter strength and benzylpenicillin yield in first promoter screen**

Carbon source for these experiments is glucose, with promoter strengths that are indicated those for growth in glucose. Blue = strains featured in Figure 2. Red = strains with low penicillin production, not featured in Figure 2.

**Single Gene Assemblies**

| Reaction | Type 2 | Type 3 | Type 4 | Pre-assembled Type 1;Type 5;Type 8 |
|---|---|---|---|---|
| Single 1 | promoter mix | pcbC | tPGK1 | ConLS;ConR1;AmpR-ColE1 |
| Single 2 | promoter mix | pclA | tENO2 | ConL1;ConR2;AmpR-ColE1 |
| Single 3 | promoter mix | penDE | tTDH1 | ConL2;ConRE;AmpR-ColE1 |

**Multigene Assembly**

| Gene 1 | Gene 2 | Gene 3 | Pre-assembled Type 6;Type7;Type 8 |
|---|---|---|---|
| Single 1 | Single 2 | Single 3 | His3;2micron;KanR-ColE1 |

**Supplementary Table 9. Golden Gate Assembly reaction parts for promoter screen to optimise conversion of ACV to benzylpenicillin and for nanopore sequencing**

| Promoter | Category | Relative Expression* |
|---|---|---|
| pTDH3 | Strong | 820 |
| pCCW12 | Strong | 575 |
| pPGK1 | Strong | 291 |
| pTEF1 | Strong | 230 |
| pHHF1 | Medium | 81 |
| pRPL18B | Medium | 60 |

**Supplementary Table 10. Promoters used in second promoter screen to optimise conversion of ACV to benzylpenicillin**

* Based on fold-change expression over background of fluorescent reporter Venus protein under each promoter, cells grown in glucose.
Taken from Lee, M.E., DeLoache, W.C., Cervantes, B. & Dueber, J.E.
ACS Synth Biol 4, 975-986 (2015).

**Single Gene Assemblies**

| Reaction | Type 2 | Type 3 | Type 4 | Pre-assembled Type 1;Type 5;Type 8 |
|---|---|---|---|---|
| Single 1 | pTDH3 | pcbC | tPGK1 | ConLS;ConR1;AmpR-ColE1 |
| Single 2 | pCCW12 | pcbC | tPGK1 | ConLS;ConR1;AmpR-ColE1 |
| Single 3 | pPGK1 | pcbC | tPGK1 | ConLS;ConR1;AmpR-ColE1 |
| Single 4 | pTEF1 | pcbC | tPGK1 | ConLS;ConR1;AmpR-ColE1 |
| Single 5 | pHHF1 | pcbC | tPGK1 | ConLS;ConR1;AmpR-ColE1 |
| Single 6 | pRPL18B | pcbC | tPGK1 | ConLS;ConR1;AmpR-ColE1 |
| Single 7 | pTDH3 | pclA | tENO2 | ConL1;ConR2;AmpR-ColE1 |
| Single 8 | pCCW12 | pclA | tENO2 | ConL1;ConR2;AmpR-ColE1 |
| Single 9 | pPGK1 | pclA | tENO2 | ConL1;ConR2;AmpR-ColE1 |
| Single 10 | pTEF1 | pclA | tENO2 | ConL1;ConR2;AmpR-ColE1 |
| Single 11 | pHHF1 | pclA | tENO2 | ConL1;ConR2;AmpR-ColE1 |
| Single 12 | pRPL18B | pclA | tENO2 | ConL1;ConR2;AmpR-ColE1 |
| Single 13 | pTDH3 | penDE | tTDH1 | ConL2;ConRE;AmpR-ColE1 |
| Single 14 | pCCW12 | penDE | tTDH1 | ConL2;ConRE;AmpR-ColE1 |
| Single 15 | pPGK1 | penDE | tTDH1 | ConL2;ConRE;AmpR-ColE1 |
| Single 16 | pTEF1 | penDE | tTDH1 | ConL2;ConRE;AmpR-ColE1 |
| Single 17 | pHHF1 | penDE | tTDH1 | ConL2;ConRE;AmpR-ColE1 |
| Single 18 | pRPL18B | penDE | tTDH1 | ConL2;ConRE;AmpR-ColE1 |

**Multigene Assebmly**

| Gene 1 | Gene 2 | Gene 3 | Pre-assembled Type 6;Type7;Type 8 |
|---|---|---|---|
| Single 1 – Single 6 | Single 7 – Single 12 | Single 13 – Single 18 | His3;2micron;KanR-ColE1 |

**Supplementary Table 11. Golden Gate Assembly reaction parts for second promoter screen to optimise conversion of ACV to benzylpenicillin**

| Strain | pcbC | pclA | penDE | pcbC promoter Strength | pclA promoter Strength | penDE promoter Strength |
|---|---|---|---|---|---|---|
| Sc.P2 | HHF1 | TEF1 | PGK1 | 81 | 230 | 291 |
| S7 | TDH3 | CCW12 | TEF1 | 820 | 575 | 230 |
| S2 | PGK1 | TDH3 | TEF1 | 291 | 820 | 230 |
| S3 | RPL18B | TEF1 | TEF1 | 60 | 230 | 230 |
| S1 | RPL18B | TEF1 | TEF1 | 60 | 230 | 230 |
| S6 | RPL18B | TDH3 | CCW12 | 60 | 820 | 575 |
| S4 | PGK1 | TEF1 | PGK1 | 291 | 230 | 291 |
| S5 | TEF1 | TDH3 | PGK1 | 230 | 820 | 291 |
| S10 | CCW12 | PGK1 | CCW12 | 575 | 291 | 575 |
| S11 | RPL18B | HHF1 | RPL18B | 60 | 81 | 60 |
| S8 | TEF1 | TDH3 | CCW12 | 230 | 820 | 575 |
| S12 | TDH3 | PGK1 | TEF1 | 820 | 291 | 230 |
| S9 | PGK1 | RPL18B | TEF1 | 291 | 60 | 230 |

**Supplementary Table 12. Promoter strengths for strains shown in Figure 3d**
